# Divergence in gene regulation at young life history stages of whitefish (*Coregonus *sp.) and the emergence of genomic isolation

**DOI:** 10.1186/1471-2148-9-59

**Published:** 2009-03-16

**Authors:** Arne W Nolte, Sébastien Renaut, Louis Bernatchez

**Affiliations:** 1 Département de Biologie, Pavillon Charles-Eugène-Marchand, Université Laval, Ste-Foy, Québec, G1V 0A6, Canada

## Abstract

**Background:**

The evolution of barriers to reproduction is of key interest to understand speciation. However, there may be a current bias towards studying intrinsic postzygotic isolation in old species pairs as compared to the emergence of barriers to gene flow through adaptive divergence. This study evaluates the relative importance of both processes in the evolution of genomic isolation in incipient species of whitefish (*Coregonus clupeaformis*) for which preliminary data suggest that postzygotic isolation emerges with intrinsic factors acting at embryo stages but also due to extrinsic factors during adult life.

**Results:**

Gene expression data were screened using cDNA microarrays to identify regulatory changes at embryo and juvenile stages that provide evidence for genomic divergence at the underlying genetic factors. A comparison of different life history stages shows that 16-week old juvenile fish have 14 times more genes displaying significant regulatory divergence than embryos. Furthermore, regulatory changes in juvenile fish match patterns in adult fish suggesting that gene expression divergence is established early in juvenile fish and persists throughout the adult phase. Comparative analyses with results from previous studies on dwarf-normal species pairs show that at least 26 genetic factors identified in juvenile fish are candidate traits for adaptive divergence in adult fish. Eight of these show parallel directions of gene expression divergence independent of tissue type or age of the fish. The latter are associated with energy metabolism, a complex trait known to drive adaptive divergence in dwarf and normal whitefish.

**Conclusion:**

Although experimental evidence suggests the existence of genetic factors that cause intrinsic postzygotic isolation acting in embryos, the analysis presented here provided few candidate genes in embryos, which also corroborate previous studies showing a lack of ecological divergence between sympatric dwarf and normal whitefish at the larval stage. In contrast, gene expression divergence in juveniles can be linked to adaptive traits and seems to be driven by positive selection. The results support the idea that adaptive differentiation may be more important in explaining the emergence of barriers to gene flow in an early phase of speciation by providing a broad genomic basis for extrinsic postzygotic isolation rather than intrinsic barriers.

## Background

The evolution of reproductive isolation is of fundamental interest in evolutionary biology because it represents a key step in speciation processes and the generation of biological diversity [1]. Merging of divergent lineages can be prevented by prezygotic barriers that reduce heterospecific mating or by decreased offspring fitness (postzygotic isolation). Some of the most inclusive studies on postzygotic isolation have focussed on taxa that have been separated for millions of years. For instance, hybrids among species of *Drosophila *are often completely sterile or inviable, which can be explained by Dobzhansky-Müller incompatibilities [2,3]. Postzygotic isolation results from genetic changes in the parental lineages that, while functional on their normal genetic backgrounds, reduce the viability or fertility when recombined in hybrids. Intrinsic postzygotic isolation is likely to manifest as soon as the respective factors are expressed, *i.e*. during early development [1], whereas effects on reproductive traits are naturally associated with the reproductive phase. Such intrinsic barriers to reproduction are thought to evolve slowly through a stochastic accumulation of genetic incompatibilities [4]. However, when young species have split only recently, extrinsic postzygotic isolation can also be effective through a more subtle effect. Alleles that reduce the fitness in a given genetic background can be removed by externally (e.g. ecological) caused natural selection. Here, heterospecific allele combinations are not lethal but perform worse than pure parental genotypes in dependence of the ecological context. Differentially adapted genes can be instrumental to generate initial patterns of genetic divergence and are thought to govern the divergence and merging of young evolutionary lineages [5-7]. At least under conditions of gene flow, speciation will be driven by natural selection imposed by external ecological factors [5,8,9] and models generally agree that intrinsic hybrid inviability is not an initial event that drives speciation [1].

There may be a bias in our perception of the contribution of intrinsic and extrinsic postzygotic isolation to speciation processes. This is because it is usually more straightforward to analyse intrinsic barriers than to grasp extrinsic barriers experimentally, since the latter will most likely depend on unknown ecological interactions. Therefore, traits that could provide a basis for genomic isolation in young lineages remain insufficiently explored. A possible approach is given by transcriptome analysis. Here, gene expression data may help identifying key genes involved in speciation since regulatory evolution is hypothesized to be a key factor in microevolutionary processes [10-12]. Genes that are regulated differently are likely to loose compatibility with the genetic environments of alternative lineages. Microarray approaches offer the potential to study genome-wide patterns of divergence, and can be considered as an inventory of characters that could serve as a basis for genome divergence. Although this does not provide evidence that selection acts on each of the particular genes under study, it will reveal the processes and functions that may be affected.

In this study, we explore by means of transcriptomics the regulatory divergence between incipient species of lake whitefish (*Coregonus clupeaformis *(Mitchill, 1818)) in order to identify candidate traits that could contribute to barriers to gene flow. This system is of particular interest to study the emergence of postzygotic isolation as the diverging lineages are of recent, most likely postglacial, origin (15 000 ya) [13]. Dwarf and normal whitefish have evolved multiple times in response to ecological selection pressures [14] and genome scans and mapping projects demonstrated that natural selection drives this divergence in multiple genomic regions [15,16] while also suggesting that the lineages are at a phase of speciation where gene flow is still occurring. On the other hand, Rogers and Bernatchez [17] have found evidence for genetic factors causing postzygotic isolation in developing eggs. The actual genes and functions involved in these processes are largely unknown due to a use of anonymous genetic markers. However, the application of transcriptome data offers a promising approach to identify candidate genes. Microarrays made for salmon (*Salmo salar, Onchorynchus mykiss*) can be readily used in whitefishes [18,19]. Derome *et al*. [20,21] and St-Cyr *et al*. [22] have identified a suite of candidate adaptive traits that display parallel changes in gene expression between adult dwarf and normal whitefish in replicated lakes.

Here, we tie in with the above studies, which suggest than both intrinsic and extrinsic barriers to reproduction play a role in the divergence of dwarf and normal whitefish at the embryo stage and during the adult life respectively. Our main objective was to compare regulatory changes at different life history stages to obtain an insight into the processes that may contribute to genomic divergence. Our results indicate that there is little regulatory divergence in embryos in sharp contrast with evidence that numerous genes display regulatory divergence in juvenile fish. Given that the latter patterns can be partially linked to ecological divergence, we conclude that extrinsic postzygotic barriers may be more important to explain early evolutionary divergence of dwarf and normal whitefish than intrinsic barriers to reproduction.

## Results

### Number and types of genes analysed

The number of features (spotted EST clones) for which we obtained gene expression data of sufficient quality for subsequent analyses was 7004 for the embryos and 5787 for the juvenile dataset. This discrepancy is correlated with technical aspects of the experiments. The number of spots that were excluded because they had a bad quality flag (obvious artefacts) after visual editing was 3209 in the juvenile dataset vs. 1055 in the embryo dataset. Furthermore, the average background in the embryo experiments was lower than in the juvenile experiments (744 vs. 849 relative fluorescence units). A total of 4293 features were common to both datasets. Accordingly, the embryo data contained 2711 and the juvenile data contained 1494 unique features. Those features of the whole embryo dataset that were associated with a GO term could be linked to 2034 unique unigene clusters and the features of the whole juvenile dataset represented 1549 unique unigene clusters.

The overall representation of gene functional groups among the expressed features between the two datasets differed significantly according to the ease score provided by the EASE software. The juvenile fish dataset contained a significant relative excess (ease score < 0.05) of unigene clusters representing three GO-Biological processes: Catabolism (55 genes, ease score = 0.004), lipid metabolism (26 genes, ease score = 0.011), proteolysis and peptidolysis (37 genes, ease score = 0.025). In contrast, the embryo dataset contained an almost significant relative excess of unigene clusters representing two GO-Biological processes: Cell cycle (44 genes, ease score = 0.095) and nucleobase\, nucleoside\, nucleotide and nucleic acid metabolism (139 genes, ease score = 0.096). These trends in the representation of genes in the two life history stages reflect the importance of metabolism and growth processes in the juvenile stage while gene transcription regulation and development predominate in the embryos.

### Genes displaying significant differences

After applying a FDR correction for multiple testing, only 33 EST clones showed significant differential expression between the embryos of dwarf and normal whitefish [see Additional file 1]. In contrast, a total of 502 EST clones displayed significant differences in gene expression between dwarf and normal whitefish in the juvenile fish dataset. This difference in the proportion of EST clones that display significant differentiation in gene expression in the two datasets was highly significant (Fisher's Exact test, p < 0.001). For the embryos, 350 out of the 7004 features tested would be expected to have false positive tests according to our significance criterion (p < 0.05). However, the number of raw significant results in the embryo analysis was 590 (8,4%). The corresponding number of significant genes in the juvenile dataset was 988 (17%), while 289 false positives would be expected. This indicates for both datasets that the number of significant tests is not explainable by the expected false positive rate. The true number of genes with differential patterns of expression was higher than the list obtained after the FDR procedure, but the trend that there was much more differentiation in the juvenile compared to the embryonic stage remains independent of the FDR procedure.

For comparisons between datasets, EST clones were assigned to EST clone groups based on; i) unigene cluster or accession numbers (latest annotation following cGRASP) unless there was no known function, and ii) unique patterns of gene expression divergence. In doing this, significant patterns were integrated over replicate clones and overrepresentation of different genes by multiple clones was corrected. Among the features displaying significant differentiation in the embryo dataset, 20 can be assigned to one of twelve unigene clusters. Eleven out of the twelve unigene clusters that display significant differentiation in the embryo dataset also appear in the list of significant unigene clusters of the juvenile dataset [see Additional file 1]. A total of 191 of the significant EST clones of the juvenile fish dataset were assigned to one of 127 unigene clusters. Accounting for the different numbers of unigene clusters that were represented by the raw data in the egg (2034) and juvenile (1549) datasets, this indicates that roughly fourteen times more genes as represented by distinct unigene clusters display overall significant differentiation in the juvenile fish than in the embryos.

### Comparisons with results from previous studies on adult fish

Some of the EST clones displaying significant divergence of gene expression in the analyses presented above have already been demonstrated to display differential expression in dwarf and normal whitefish. Only one EST clone for which significant differentiation was detected in the embryo dataset (CA057378; Accession AY872256; *Oncorhynchus mykiss *IgH.A locus) was previously found to be differentially expressed in white muscle between laboratory dwarf and normal whitefish [21]. In contrast, 108 EST clones that can be assigned to 44 different EST clone groups (identical accessions and unique patterns of expression) identified in whole juvenile fish [see Additional file 2] also show significant differentiation in gene expression in white muscle of adult fish of the same dwarf and normal strains in a controlled common environment [21]. Although different tissues and life history stages were compared, there was a significant excess of 31 out of 44 EST clone groups (Fishers' test, p = 0.0403) where gene expression divergence between dwarf and normal whitefish was congruent in the pattern of up or down regulation in dwarfs relative to normals [see Additional file 2] as compared to a random distribution of changes in both directions. Likewise, a comparison of the set of EST clones displaying significant gene expression divergence in juvenile fish reveals matches with candidate features that have been identified in independent natural lakes [20,22]. It should be noted that the study by Derome et al. [20] used a less inclusive microarray containing five times less features, which reduces the relative power of that study to identify genes that were found here or by St-Cyr *et al*. [22]. A total of 96 EST clones that displayed significant divergence in whole juvenile dwarf and normal whitefish of the strains studied here could be matched with EST clones displaying parallel adaptive divergence in gene expression in liver or white muscle of adult dwarf and normal whitefish from Cliff Lake (Maine, USA) and Indian Pond (Maine, USA) [see Additional file 3]. Ten out of 26 different EST clone groups (grouped as described above.) show regulatory changes in the controlled environment that were congruent with the patterns of candidate adaptive traits as observed in adult tissues in natural lakes. Among these, there appears to be a bias in that eight of ten affected genes are related to energy metabolism, which shows that regulatory changes between dwarf and normal whitefish related to this function are more constant across different environments, life history stages and tissues than those related to other functions [see Additional file 3].

In contrast to the observations for juvenile and adult fish of the experimental strains in controlled common environment [see Additional file 2], the direction of upregulation vs. downregulation of the transcript level of dwarf whitefish relative to normal whitefish shows less congruence when samples from natural environments and a new tissue (liver) are included in the comparison. Thus, 16 out of 26 groups of features that display significant divergence of gene expression in candidate adaptive features differ in the direction of the change between juveniles and adults (16) or between adult muscle and liver tissue (3) [see Additional file 3].

## Discussion

Our results revealed a pronounced pattern of gene expression divergence for 502 EST clones between 16-week old juvenile dwarf and normal lake whitefish (*Coregonus clupeaformis *complex) as compared to embryos of the same experimental groups, which displayed little divergence in gene expression (33 EST clones). Although the number of evolutionary changes causing the observed differences is currently unknown, a fourteen-fold excess of unigene clusters displaying significant differentiation in the juvenile dataset suggests that multiple regulatory changes take effect only after development has passed the embryo stage. If gene expression divergence were the result of random accumulation of evolutionary differences between the studied populations, roughly equal proportions of gene expression differences would be expected to occur in both life history stages. The much more likely scenario is that evolutionary change in gene expression plays a greater role at the juvenile stages than the embryonic stage.

The general pattern observed for gene expression divergence and regulatory changes resembles the ontogeny of morphological features across the animal kingdom. Briefly, early developmental stages are usually extremely well conserved, whereas adult phenotypes vary as a consequence of evolutionary divergence, a classical observation made by early developmental biologists [23,24]. This observation has often been made for distantly related taxa. This study suggests that the same evolutionary pattern may not only apply to morphological characters, but also to transcriptomic divergence at the level of recently evolved lineages of fish. Below, the observed changes in gene expression are discussed in relation to life history divergence of dwarf and normal whitefish. We propose that the excess of gene expression divergence in juvenile fish relative to embryos can be attributed to selective pressures that are related to ecological adaptation in the juvenile and the adult phase rather than an evolutionary constraint on divergence in embryonic stages.

A key concern in the analyses was that the absence of differentiation observed in the embryos could represent an artefact. Developmental processes and therefore gene expression in embryos can be expected to change rapidly throughout ontogeny. The problem such heterogeneity in gene expression imposes for the analysis resembles that of allometry in studies of body shape [25] and the relevance of heterogeneity for the analysis of gene expression data has recently been pointed out by Leek and Storey [26]. If different stages with accordingly changed patterns of gene expression were sampled, the variance in gene expression would be inflated. Intra group variance could then exceed the between group variance to a point where the latter is not detected as significant in ANOVA based statistical approaches. The extent to which such variation occurs in the transcriptome can be inferred from a study by Arbeitman *et al*. [27] who performed a very complete analysis of patterns of gene expression throughout development of *Drosophila melanogaster *and found that significant heterogeneity was observed for 52% of all studied genes during the embryogenesis but few genes displaying developmental heterogeneity in adult *Drosophila*. Moreover, some classes of genes that are expressed during the segmentation phase of fishes show highly dynamic and cyclical patterns of expression through short periods of time [28]. The sampling of a relatively well-defined segmentation stage as done in this study is merely a snap-shot of the whole embryogenesis and should therefore contain considerably more genes with relatively constant expression patterns that are consequently useful for ANOVA. Accordingly, the inter sample variance in gene expression for eggs and juveniles was in the same order of magnitude (mean inter-sample variance 0.0085 and 0.0066 respectively) as estimated from a more inclusive dataset of the same stages including technical replication (Renaut *et al*. unpublished). Even if only half of all genes (comp. above; [27]) in the embryo dataset displayed homogeneous gene expression, one would still expect to detect considerably more significant genes if the proportion of significant genes in the embryo data was identical to that in the juvenile data. Although an effect of developmental heterogeneity cannot be excluded, it is unlikely to explain the observed excess in gene expression divergence in the juveniles.

### The ontogeny and evolution of gene expression differentiation

Dwarf whitefish from Lake Témiscouata and normal whitefish from Lake Aylmer studied here have a similar reproductive biology. Adult fish live in the lake throughout the year and enter tributaries only for a brief spawning period. Spawning migrations are short and occur on a daily basis at night from late October to early November. Eggs are dispersed in currents and settle into rock and gravel substrates where they are left unattended to develop. Ninety-one percent of the unigene clusters that displayed significant regulatory divergence in the embryos were also significantly divergent in juvenile fish and in one case, found to be differentially expressed in white muscle tissue of adults of the same laboratory strains used here. However, none of the significantly different genes of the embryo stage was found to be a candidate for adaptive divergence in previous studies. The fact that ecological divergence of the egg stages of whitefishes has not been discovered to date together with the relative lack of gene expression differentiation suggests strongly that little or no adaptive evolutionary divergence has occurred specifically at the embryo stage and that most of the gene expression differentiation between dwarf and normal whitefish must develop at a later phase of the ontogeny.

Upon hatching whitefish larvae are washed from their natal river into the lakes were they spent their entire life. To date it is unknown at which phase of the life history the ecological differentiation into the dwarf (limnetic) and normal (benthic) lifestyles occurs in nature. In a study of dwarf and normal whitefish in Cliff Lake, larvae of dwarf and normal populations did not differ in their hatching time, diet, distribution and vertical migration within the lake [29]. Unlike their parents, the larvae lived in total syntopy and there was no evidence for differential trophic ecology or circadian vertical migration, suggesting that ecological divergence of the two forms must begin after the larval stage [29]. The experimental populations used in this study had a total age of 16 weeks and had morphologically transformed into juvenile fish (development of finrays and scales) for approximately 8 weeks before sampling was done. At the level of the transcriptome, the transformation into the juvenile stage is accompanied by an emergence of gene expression divergence between the two forms that was absent at the embryo stage. Also, gene expression divergence at this juvenile stage could be matched with patterns observed in adult fish. 108 EST clones representing 44 differentially regulated genes or accessions [see Additional file 2] also displayed regulatory divergence in muscle tissue of adult fish belonging to the same experimental groups and kept in a controlled common environment [21]. There was a significant excess of congruent regulatory change, which suggests that gene expression divergence between dwarf and normal whitefish is of a similar nature in juvenile and adults. The notion that regulatory divergence does not change much after the development of the adult morphology has finished is in line with the observation that many patterns of gene expression change little throughout adult life in *Drosophila *[27]. Hence, the juvenile stage studied here is useful to study the transition of life histories from non-differentiated larval fish [29] to adult dwarf and normal whitefish with pronounced differential adaptation [13].

A total of 96 of the EST clones identified here matched with 26 accessions or genes that were also described by Derome *et al*. [20] and St-Cyr *et al*. [22] [see Additional file 3] who found a recurrent association of divergence in gene expression and parallel adaptive differentiation in multiple lakes (including Cliff lake) for these genes. Briefly, dwarf whitefish tend to have a shorter lifespan and begin to reproduce earlier than normal whitefish. They are specifically adapted to the open water where they specialise on a zooplankton diet as opposed to the more benthic lifestyle of normal whitefish [13]. According to Trudel *et al*. [30] this ecological differentiation is driven by differences in metabolic rate and energy allocation between dwarf and normal whitefish. Derome *et al*. [20] and St-Cyr *et al*. [22] have inferred candidate adaptive traits bases on patterns of parallel divergence in independent lake systems each containing dwarf and normal whitefish. In agreement with the experimental results of Trudel *et al*. [30], the biological function of a part of these candidates implies a role in energy metabolism. Our results on the gene expression divergence between juvenile dwarf and normal whitefish has revealed ten out of 26 genes which match adaptive regulatory changes in the adult stage irrespective of the fact that different tissues were used. Most conspicuously, eight of these ten genes can be associated with energy metabolism. The comparison of different studies reveals a recurrent pattern of up regulation of transcripts for Glyceraldehyde-3-phosphate dehydrogenase, Fructose-bisphosphate aldolase A, Beta-enolase and Trypsin-1 precursor as well as a down regulation of transcripts for a mitochondrial precursor of Cytochrome c oxidase polypeptide VIa, Nucleoside diphosphate kinase and Nucleoside diphosphate kinase A in dwarf relative to normal whitefish [see Additional file 3] in different tissues and life history stages. This adds evidence for the hypothesis that energy metabolism as a complex trait plays an ubiquitous role in driving the adaptive divergence between dwarf and normal whitefish while other candidate adaptive traits play more tissue and context specific roles.

The results obtained here for juvenile fish and the comparison of the direction of the regulatory changes in different tissues or the controlled common environment vs. natural lakes shows that regulatory changes at candidate adaptive traits are not always congruent in terms of the direction of the change. It must be emphasized though, that the inference of adaptive divergence from parallel regulatory changes relies on the use of comparable samples. If tissue type and environmental context vary, the regulatory response can be expected to vary as well. Accordingly, there was more agreement in the direction of the regulatory changes when comparison based on liver tissue or natural environments were excluded [see Additional file 2]. This implies that the inference of an adaptive value of parallel divergent traits by Derome *et al*. [20] and St-Cyr *et al*. [22] is not invalidated by contrasting patterns of regulatory divergence for a given candidate gene in juvenile fish and vice versa. Although the relationship of the direction of regulatory change according to tissue context, age and environmental factors needs to be addressed in future studies, the fact remains that many candidate genes for adaptive divergence in adult fish also show regulatory differentiation at the juvenile stage. In contrast to the embryo stage analysed here, these results suggest that the divergence in gene expression in juvenile fish is subject to directional selection related to adaptive divergence.

The identification of candidate adaptive gene expression divergence still draws an incomplete picture of the processes that ultimately lead to the life history differentiation into dwarf and normal whitefish. If adaptive differences at the transcriptome level are expressed as early as young (16 weeks) juvenile stage, then it is likely that these genetic factors may initiate the development of life history divergence. Although data on juvenile fish from natural lakes are missing, inferences can be made from our laboratory populations. Experimental fish were kept in a controlled common environment and the candidate adaptive traits remain prevalent at the transcriptome level suggesting that juvenile fish already display differential adaptation. On the other hand, the phenotypes of our experimental populations bred in the laboratory seem to contradict a persistent ecophenotypic differentiation between dwarf and normal whitefish. Although morphological features distinguishing dwarf and normal ecotypes are heritable [16], the differentiation of life histories under laboratory conditions is less pronounced than in nature. Dwarf whitefish remain only slightly smaller than normal whitefish and grow older than their natural counterparts (unpublished observation). This strongly suggests that there is an environmental component that interacts with a genetic one to shape the ecophenotypic differentiation of dwarf and normal whitefish.

### A potential role for stabilizing selection

Aside directional selection driving divergence at the juvenile and adult stages, an alternative explanation for the relative lack of embryo gene expression may be derived from developmental biology. The embryos during the segmentation phase that were studied here correspond closely to the phylotypic stage, a developmental phase that corresponds to an archetype bauplan of all representatives of a given phylum. This stage is generally extremely conserved [31] suggesting that strong evolutionary constraints prevent divergence. While the morphological conservation of phylotypic stage embryos remains undisputed, it has been found that patterns of gene expression need not be constrained. Selection on adult genotypes may alter gene regulation in embryos [32] and developmental system drift [33], a process whereby gene regulatory networks evolve without changing the expression level of a gene, have been demonstrated for even closely related taxa [34]. This casts doubt on the validity of the phylotypic stage concept at the transcriptome level [34]. Given these alternatives, the similarity between embryos of dwarf and normal whitefish could imply the action of strong evolutionary constraints that preserve identical patterns of gene expression relative to what we observed at the juvenile stage. Under an evolutionary constraints hypothesis, genes that determine the expression level of a transcript may still evolve as long as the sum of their effects would not be changed. F1 – hybrids or segregating backcrosses would prove useful to reveal such cryptic regulatory divergence. They would combine the altered regulatory elements into a common genetic background, which can result in misexpression of genes [35-37]. Very much in line with this, crossing experiments by Lu and Bernatchez [38] and Rogers and Bernatchez [17] suggest that F1 hybrids or backcrosses of the same populations studied here suffer from raised embryonic mortality in the same stage studied here. This would indicate the presence of alleles or genes that malfunction and should manifest as misexpression at the transcriptome level (Renaut et al. *in prep*). In any case, the scarcity of regulatory divergence observed here for the embryo stage suggests that regulatory divergence is rare and may have a narrow genomic basis involving only a small number of genetic factors. This view would agree with the fact Rogers and Bernatchez [16] have only found a limited number of QTL associated with hybrid embryo mortality in their mapping analysis.

### Implications for the emergence of genomic isolation and speciation in whitefish

At the level of the transcriptome, only traits causing regulatory divergence produce a phenotype that differs between two diverging lineages. These could be affected by natural selection and are therefore candidate traits that may reduce the fitness of individuals of mixed ancestry. Thus, a screen for regulatory divergence can identify traits that could have fitness effects under conditions of gene flow and serve as a genetic basis for the emergence of genomic isolation. Models suggest that recombination can oppose genomic divergence and speciation because loci that are not selected for may still be exchanged relatively freely among populations [5,39]. Even if single genetic factors have a strong isolating effect, they may not be sufficient to reduce gene flow throughout the genome if hybrids are partially viable, as it is the case in *Coregonus *[17,38]. It is therefore evident that the more loci are divergent between two lineages, the easier it becomes to explain how these could, as a whole, provide a basis for evolutionary divergence and reproductive isolation. Few transcripts are regulated differently at the embryonic stage analysed here, which points towards a comparably small number of genes that may be differentially regulated between dwarf and normal whitefish embryos, at least relative to later stages. Although Rogers *et al*. [17] provided evidence for the presence of genetic factors causing considerable hybrid embryo mortality, it may be concluded that the regulatory changes that could cause postzygotic isolation at the embryo stage are comparably rare. Admittedly, the question to which degree this would be sufficient to prevent gene flow in nature cannot be answered at present. In any case, it would be easier to explain a reduction in gene flow if there was a broad genomic basis to reproductive isolation, i.e. effects of multiple traits that could be selected for, rather than few. Our results suggest strongly that the largest part of regulatory divergence occurs throughout the juvenile and adult phases. A pattern of more pronounced divergence in juvenile and adult fish corresponds well with what has been observed in studies on interspecific regulatory divergence [35]. Moreover, a suite of traits contributing to a diversified adult phenotype is expected to play a more important role in evolutionary divergence as compared to genes producing the conserved phylotypic stage phenotype [40].

A gap in the sampling of potentially relevant life history stages of this study is that the reproductive phase could not be analysed here. Studies on *Drosophila *[36] and *Mus *[41] have demonstrated an above average rate of regulatory divergence in genes associated with reproduction and in addition, have shown that these genes may be among the first to cause reproductive isolation. Future studies will have to show whether reproductive characters may play the same crucial role in evolutionary divergence of incipient species of fish or if ecologically selected traits are among the first to cause genomic isolation.

## Conclusion

If the relative rarity of gene expression divergence in embryo stages was a general pattern in recently diverged species, then a focus on embryo dysgenesis in studies of the early evolution of postzygotic isolation could be misleading in that they would distract from a large pool of adult characters. While tests for embryonic mortality are straightforward, it is much more difficult to test the role of particular genes on complex adult phenotypes as those may have small effects [42] or their effects may depend on unknown environmental components. Still, the transcriptomic patterns in whitefish suggest that a full understanding of how gene flow is reduced among incipient species may depend more heavily on genes affecting adult phenotypes than developmental phenotypes. In agreement with this, van der Sluijs *et al*. [43] suggest, for closely related cichlids from Lake Victoria, that postzygotic reproductive isolation is mediated by extrinsic selection rather than intrinsic hybrid dysgenesis. Furthermore, studies across a broader phylogenetic range of taxa show that intrinsic genomic incompatibility evolves slowly and after the point of speciation between diverging species [44,45]. Our results together with these studies support the view that subtle selective pressures and ecological interactions that are related to specific complex environments may be the key in explaining incipient genomic divergence [5,43,45].

## Methods

### Strains, crosses and fish maintenance

Eggs of *Coregonus clupeaformis *were obtained from lab strains kept at the LARSA (Laboratoire de Recherche en Sciences Aquatiques, Université Laval) or harvested from wild fish that were caught on their natural spawning grounds. Normal whitefish used here originate from Lake Aylmer (Basin of the St. Lawrence River, southern Quebec) and were sampled at the spawning site in the St. Francois River in Disraeli (45° 54'N, 71° 20'W) in 1996 (as detailed in Lu and Bernatchez, 1998). Since then, they were kept in the laboratory as an outbred lab strain. Dwarf whitefish originate from Lake Témiscouata (St. Johns' river system in southern Quebec) and were caught on their spawning grounds in the Touladi River (47° 41'N, 68° 47'W). Like the normal whitefish, dwarfs were maintained as an outbred laboratory strain. We also included new wild caught material from Lake Témiscouata dwarf whitefish collected in October 2006.

Eggs and semen were stripped from deafened fish, fertilized *in vitro *and incubated on grids that were submerged in slowly flowing water of a temperature of 4,5–5,5°C. All egg batches were incubated in the same flow through system and were thus subjected to compartments of the same environment. Weekly treatments with malachite green oxalate were performed to inhibit growth of fungi. Morbid eggs or embryos were removed on a daily basis. After hatching, free-swimming larvae were transferred into aquaria (50 × 25 × 30 cm) and fed *ad libitum *with *Artemia *nauplii and complemented with commercial fish food (Epac CW 4/6, Epac CW 6/8; INVE AQUACULTURE Inc., Salt Lake City, Utah, U.S.A.). All aquaria were aerated and connected by a flow through and filtering system that fed each aquarium from a common pool. This permitted constant water exchange and near identical temperature and chemical conditions. The temperature in the rearing tanks was kept at 8°C for the first 8 weeks, raised to 10°C for 3 weeks and finally adjusted to 12°C.

To capture the within population variance in gene expression and reduce family specific effects, we generally used crosses that were composed of several parents depending on the availability of mature fish at a given time. We have created two independent experimental groups for each biological group (dwarf, normal) studied here. The group DD-E was derived from the lab strain of the dwarf whitefish from Lake Témiscouata and was created using one female and five different males. DD-G was created by crossing wild caught dwarf whitefish (leg. Nolte, Renaut and Bernatchez, 25^th ^Oct. 2006) from the same lake using multiple females and multiple males. Two groups of normal whitefish (NN-C and NN-I) were created from one and five as well as two and three females and males of the lab strain of normal whitefish from Lake Aylmer, respectively.

### Stages and samples

It was our goal to analyse gene expression in a developmental stage corresponding to the phase for which Lu and Bernatchez [38] and Rogers and Bernatchez [17] observed increased embryo mortalities. We found that this corresponds to the beginning of the segmentation period. In this phase of development, an anterior-posterior axis has developed and undergoes divisions into body segments (for a detailed account on phases of fish development see [46]). Progress of development in this phase can be evaluated by counting body segments, which are added successively. This task can be performed on live eggs with the help of a binocular. Due to slight batch-to-batch variation in the precise timing of the segmentation process, we assessed developmental stages of embryos entirely by morphological features, rather than age. In our experiments, the process of segmentation began after roughly 16 days of development and ended after 29–31 days. Embryos were examined once or twice daily, from the moment that the tail bud of the embryo detached from the yolk sac (at day 20–22). It was easier to count only those segments in the part of the tail that was detached rather than all segments of the live embryo within the intact eggs. For our experiments we chose embryos that had formed approximately 20 segments in the detached portion of their tail. This stage appeared to be relatively easy to identify as at the same time the tail started moving and bent to an angle of approximately 30° (tail curvature). Furthermore, in this stage the optic primordium begins to hollow thus initiating the formation of the lens in the eye. This developmental stage corresponds best with the 20–25 somite stage observed in *Danio rerio *after only 19 hours of development at 28.5°C (compare ) [46]. Eggs that were chosen for experiments were individually examined using binoculars. Only apparently viable eggs with well-formed embryos were used. In these, the number of segments was counted through the chorion. This introduces some uncertainty in the somite count but preserves intact eggs and embryos for RNA extraction. Whole eggs were preserved in RNA later (Ambion) and frozen at -20°C for storage.

Juvenile fish were chosen as the next sampling stage as these represent an immature adult phenotype. All hatched larvae were transferred to basins and started external feeding in mid January 2007. The larvae had developed fin rays by the end of January 2007. We sampled juvenile fish at an age of approximately 16 weeks (May 10^th ^2007), when these attained a weight of approximately 800 mg (540–1190 mg). At this stage, the development of morphological features is finished and the young whitefish resemble their parents. Individuals chosen for gene expression analysis were well developed and in good general shape (vs. slow growing and meagre, as observed in some specimens). Sampling was done in the morning following an 18 hour fast. Fish were then sacrificed with a blow, kept on ice and homogenized in TRIzol reagent for RNA extraction as quickly as possible (waiting time no longer than 20 min). The homogenate was stored at -80°C prior to RNA extraction.

### Experimental design and choice of samples

The gene expression analysis for this study focuses on the divergence at different life history stages of dwarf and normal whitefish. Eight pairwise (dwarf vs. normal) comparisons for both the embryonic and the juvenile fish stage were performed resulting in two sets of eight microarrays per stage (see Table 1). Initial testing had shown that even the more sensitive Gene Array 350 Kit (see below) ideally requires 5 μg of total RNA per sample and experiment. Given that only 2,5 – 3 μg of total RNA could be extracted from a single embryo, pools containing the total RNA of five embryos were used for the embryo experiments. This pooling approach integrates patterns of gene expression over a larger number of individuals but would nevertheless reveal differences between group means as tested for in an analysis of variance. Juvenile fish extractions yielded large quantities of total RNA and were used individually.

**Table 1:** Experimental design and types of biological samples used in microarray experiments.

**Pairwise comparison**	**Dwarf whitefish experimental groups and properties of samples**		**Normal whitefish experimental groups and properties of samples**	
1 Embryo	18 segments, tail curved up to 30°	DD-E	20 segments, tail curved up to 30°	NN-C
			
2 Embryo	23–25 segments, tail curved up to 30°.		20 segments, tail curved up to 30°	
			
3 Embryo	18 segments, tail curved up to 30°		20 segments, tail curved up to 30°	
			
4 Embryo	18 segments, tail curved up to 30°		20 segments, tail curved up to 30°	

5 Embryo	15–20 segments in detached tail.	DD-G	tail partially segmented, curved up to 30°.	NN-I
			
6 Embryo	15–20 segments in detached tail		tail partially segmented, curved up to 30°.	
			
7 Embryo	15–20 segments in detached tail.		tail partially segmented, curved up to 30°.	
			
8 Embryo	10–20 segments in detached tail.		tail partially segmented, curved up to 30°	

9 Juvenile	1.04 g	DD-E	1.06 g	NN-C
			
10 Juvenile	0.96 g		0.63 g	
			
11 Juvenile	0.96 g		0.89 g	
			
12 Juvenile	0.84 g		1.19 g	

13 Juvenile	0.99 g	DD-G	1.03 g	NN-I
			
14 Juvenile	0.54 g		0.85 g	
			
15 Juvenile	0.91 g		0.78 g	
			
16 Juvenile	0.80 g		0.87 g	

Analysis of gene expression was performed pair wise for a set of embryo experiments (1–8) using pools of 5 eggs while single juveniles were used in experiments 9–16. Staging of embryos and juveniles is assessed by key developmental parameters as described in the text. For embryos the number of somites in the part of the tail that is detached from the yolk as well the observed tail curvature is given. The number of somites is an approximation as it was determined *in vivo*. For juvenile fishes we provide the total body weight in gram.

The same representation of experimental groups was used in both the embryo and juvenile fish experiments. Thus four replicates used samples from the groups DD-E and NN-C (see Table 1). Tests for differentiation among groups require that there is a homogeneous distribution of traits within groups. However, transitory developmental stages, by their very nature, change constantly which may introduce biases when the sampling is not balanced. In order to reduce artefacts a set of samples that are similar with respect to developmental features (segment count) was chosen (Table 1). The sampling of juvenile fish is less difficult as they have finished their morphogenesis. Their ontogeny is also slower and probably reduced to relatively constant growth processes. Juvenile fishes were chosen to represent a similar body mass range (Table 1).

### Analysis of gene expression

Total RNA was extracted using the TRIzol Reagent (Invitrogen) according to the protocol of the vendor. For the embryo experiments, five whole embryos preserved in RNAlater were homogenized using a bead mill (Quiagen) while for the juvenile fish experiments a single whole juvenile fish was homogenized using a polytron homogenizer. Crude total RNA was further cleaned by ultra filtration using microcon (Millipore) spin columns (embryo experiments) or a combination of a lithium chloride precipitation (addition of 1 Volume 5 M LiCl, incubation at -20°C for 2 hours, centrifugation at 16.000 g at 4°C for 30 min, final wash of the resulting pellet in 70% ethanol) and subsequent ultra filtration (juvenile fish experiments). Total RNA was quantified and quality checked using the Experion™ RNA StdSens Analysis Kit (BIO RAD). Total RNA was stored in pure water supplemented with Superase-In™ RNase Inhibitor (Ambion) at -80°C.

Gene expression analysis was performed using the 16 K v2.0 Salmon cDNA microarray as provided by the cGRASP consortium (von Schalburg *et al*. 2005). This microarray comprises 16 006 different cDNA features derived from salmonids as well as control spots that have previously been demonstrated to be useful to analyse gene expression in whitefish [20-22]. In each experiment, two different RNA samples are transcribed into strands of cDNA that are end-labelled with a specific oligonucleotide sequence. Samples are co hybridised to the microarray and the relative quantity of the hybridised product is assessed via fluorescent detection reagents that are specific to the end labels of a given sample. These experiments were made using the Genisphere 3DNA Array Detection Array 350™ Kit (Cy3/Alexa647) and Genisphere 3DNA Array Detection Array 50™ Kit (Cy3/Cy5) for the embryos and juveniles respectively and followed the protocols of the vendor. Dyes were swapped between different pairwise comparisons. Per sample and slide, we have used approximately 4–5 μg of total RNA in the embryo experiments and 18–20 μg of total RNA in the juvenile fish experiments. Reverse transcription reactions were performed using the Superscript II Kit (Invitrogen).

Microarrays were scanned using a ScanArrayTM Express scanner (Packard Bioscience) and quantified using the Quantarray software. The positioning of all grids was checked manually for both dye channels. Suspicious spots of inconsistent shape or obvious artefacts were marked with a bad quality flag. Raw data were quantified using the histogram method and exported into text files. Microarray data are deposited at ArrayExpress, a public repository, under the following experiment accession number (ArrayExpress accession: E-MEXP-1973). Input for the statistical analyses was generated from separate text files using a Perl script. For each spot, local background was subtracted from the PMT value. Data were subsequently used in the statistical analyses only if there was no more than 12,5% missing or unusable data per gene (e.g. a single value in a series of eight pairwise comparisons). Here, unusable may mean: i) a bad quality flag or ii) a gene expression value that is lower than the average background + 2 times its standard deviation for both samples measured on a given spot. The average background was determined from 800 empty spots or blank wells on the 16 K salmon chip, provided that these spots were not excluded due to artefacts after visual inspection. Statistical analysis of the data was performed in R version 2.6.1 (The R Foundation for Statistical Computing Copyright (C) 2007 ISBN 3-900051-07-0) using the R package R/maanova Version 1.4.1 [47,48]. Raw data was imported and missing data were imputed (KNN method, 10 nearest neighbours). Data was log2 transformed and normalized using the lowess algorithm. An ANOVA model including the following terms as fixed sources of variance: Type (population, the term of interest), Dye (Fluorescent dye) and Sample (biological sample) while Array (individual microarray) was included as random term. Statistical testing for divergence in gene expression between groups is based on an F test (Fs test option in R/maanova). P-values were determined by comparing observed values to a distribution obtained by randomly shuffling values of samples (1000 permutations). The FDR procedure as implemented in R/maanova was used to correct for multiple testing using an FDR cut off value of 5%.

The populations studied here have previously served to study gene expression divergence at the adult stage between specific tissues of dwarf and normal whitefish using a similar ANOVA. EST clones or genes for which significant differentiation was found in this study were compared with lists of candidate genes from studies on white muscle and liver tissue [20-22]. This comparison was based on EST clone ID numbers for comparisons with the studies that used the same microarray [21,22]. Derome *et al*. [20] used an earlier and less inclusive salmon microarray. In order to match results with the current dataset, EST clone sequences of candidate genes described by Derome *et al*. [20] were compared to all features on the 16 K v2.0 Salmon cDNA microarray using the BLAST algorithm [49] as implemented in BioEdit [50]. All sequences could be unequivocally matched to features on the new arrays based on the longest 100% identical sequence. The annotation of all features reported here follows the latest version of the gene annotation file for the 16 K Salmon microarray (as of Feb 13^th ^2008) provided on the cGRASP homepage . Analyses of the representation of functional categories of genes among datasets was based on the DAVID/EASE programs [51].

## Authors' contributions

AWN conceived the study, collected the data, performed the analyses and wrote the manuscript. SR contributed to all parts of the experimental design, data collection and analysis. LB conceived the study, coordinated the work and edited the manuscript. All authors read and approved the final manuscript.

## Supplementary Material

Additional file 1 **EST clones for which significant divergence was detected between embryos or juveniles of dwarf and normal whitefish.** List of all EST clones for which significant divergence of gene expression was detected for dwarf and normal whitefish (33 and 502 EST clones for the Embryo and Juvenile datasets). EST clone and annotation, the Log2-fold change of dwarfs relative to normals and the FDR adjusted Permutation p-value (significance criterion in this study) are given. Click here for file

Additional file 2 **Regulatory changes between juvenile dwarf and normal whitefish in a common environment.** Similarity of regulatory changes between dwarf and normal whitefish at different life history stages in controlled common environments. All comparisons are based on the same strains (Lake Témiscouata "dwarf" and Lake Aylmer "normal" respectively). 108 EST clones that display significant differentiation in gene expression in whole juvenile fish (this study) were previously found to be differentially expressed in muscle tissue from adult fish [21]. When genes are represented by several EST clones these are grouped according to their annotation (accession number and gene name) and patterns of gene expression. "up" or "down" regulation describes the direction of the change of gene expression in the dwarf whitefish relative to normal whitefish. Biological functions are as given in Derome et al. [21]. EM = energetic metabolism; IR = immune response; OB = oxygen binding; OF = other function; PS = protein synthesis; RPD = reproduction; MCR = muscle contraction regulation; PM = protein metabolism. Despite the fact that different tissues were used, 31 out of 45 genes show congruent directions of significant regulatory changes in juvenile and adult fish (last column), which points towards ubiquitous patterns of expression divergence at juvenile and adult stages in a common laboratory environment. Click here for file

Additional file 3 **Regulatory changes between juvenile dwarf and normal whitefish at candidate adaptive traits.** Comparison of significant regulatory changes between juvenile dwarf and normal whitefish with patterns observed at candidate adaptive traits. Derome et al. [20] and St-Cyr et al. [22] analysed gene expression in white muscle and liver tissue of adult fish from species pairs in natural lakes (dwarfs and normals from Cliff Lake and Indian Pond) and identified candidate adaptive traits based on patterns of parallel divergence. 96 EST clones that match candidate adaptive traits displayed significant patterns of divergence in whole juvenile dwarf and normal whitefish in this study (Lake Témiscouata "dwarf" and Lake Aylmer "normal" respectively). Column contents correspond with Additional file 2 [see Additional file 2], with an additional column describing regulatory changes found by St-Cyr et al. [22] for adult fish liver tissue and the following additional biological functions as described in St-Cyr et al. [22]: DT = detoxification; LM = lipid metabolism; BT = blood and transport; GLF = germ-line formation; PD = Protein degradation. A minus (-) indicates that gene expression divergence was not tested or detected in the corresponding adult tissue. Ten out of 26 genes clones show regulatory changes in the controlled environment that are congruent with the patterns observed in candidate adaptive traits in independent dwarf normal pairs in natural lakes. Eight of these ten genes are related to energy metabolism, which shows that regulatory changes between dwarf and normal whitefish related to this function are more constant across different environments, tissues and life history stages than those related to other functions. Click here for file
