# Supplementary material for: Divergence in gene regulation at young life history stages of whitefish (Coregonus sp.) and the emergence of genomic isolation
Source: BMC Evol Biol. 2009 Mar 16;9:59. doi: 10.1186/1471-2148-9-59 (PMC2662803; doi:10.1186/1471-2148-9-59)
Supplement: Additional file 2 — Regulatory changes between juvenile dwarf and normal whitefish in a common environment. Similarity of regulatory changes between dwarf and normal whitefish at different life history stages in controlled common environments. All comparisons are based on the same strains (Lake Témiscouata "dwarf" and Lake Aylmer "normal" respectively). 108 EST clones that display significant differentiation in gene expression in whole juvenile fish (this study) were previously found to be differentially expressed in muscle tissue from adult fish [21]. When genes are represented by several EST clones these are grouped according to their annotation (accession number and gene name) and patterns of gene expression. "up" or "down" regulation describes the direction of the change of gene expression in the dwarf whitefish relative to normal whitefish. Biological functions are as given in Derome et al. [21]. EM = energetic metabolism; IR = immune response; OB = oxygen binding; OF = other function; PS = protein synthesis; RPD = reproduction; MCR = muscle contraction regulation; PM = protein metabolism. Despite the fact that different tissues were used, 31 out of 45 genes show congruent directions of significant regulatory changes in juvenile and adult fish (last column), which points towards ubiquitous patterns of expression divergence at juvenile and adult stages in a common laboratory environment. [file 1471-2148-9-59-S2.doc]

| **EST clone ID** | **Accession Number** | **Gene** | **Biological Function** | **Juvenile fish (whole)** | **Adult Muscle**  **[21]** | **Congruence of regulatory changes** |
| --- | --- | --- | --- | --- | --- | --- |
| CB496806; CA042792 | O13085 | Cytochrome c oxidase polypeptide VIa, mitochondrial precursor | EM | down | down | yes |
| CA057166; CB492725; CA041894; CB494346; CB509700 | P15429 | Beta-enolase | EM | up | up | yes |
| CB491722; CB497381; CB493362; CB497649 | P19804 | Nucleoside diphosphate kinase B | EM | down | down | yes |
| CB498538 | P70083 | Sarcoplasmic/endoplasmic reticulum calcium ATPase 1 | EM | up | up | yes |
| CB510934; CB492030; CB511030; CA042095; CB492512; CB510537; CB497378; CB493401 | Q05982 | Nucleoside diphosphate kinase A | EM | down | down | yes |
| CB499941; CK991017 | Q36860 | Cytochrome c oxidase subunit 3 | EM | down | down | yes |
| CN442519 | Q37677 | Cytochrome c oxidase subunit 2 | EM | down | down | yes |
| CB511422 | Q50KA9 | Nucleoside diphosphate kinase A | EM | down | down | yes |
| CA054312; CB518099; CA056815 | Q92122 | Pyruvate kinase muscle isozyme | EM | up | up | yes |
| CB492944 | Q9DAK9 | 14 kDa phosphohistidine phosphatase | EM | down | down | yes |
| CN442555; CN442514; CK991178; CN442543; CA045984 | Q9ZZM6 | Cytochrome c oxidase subunit 1 | EM | down | down | yes |
| CA051720 | AB162342 | Oncorhynchus mykiss genes, MHC class I a region, complete and partial cds | IR | down | down | yes |
| CB497373; CB496526; CA039335 | P68246 | Troponin I, fast skeletal muscle | MCR | down | down | yes |
| CK990545; CA042157; CK990626; CA061048; CA043324; CK991314; CB501401 | O42197 | Beta-2-microglobulin precursor | OB | up | up | yes |
| CB510619; CB496826 | AJ272369 | Oncorhynchus mykiss mRNA for type II keratin E1 (E1 gene) | OF | down | down | yes |
| CA057378 | AY872256 | Oncorhynchus mykiss IgH.A locus, partial sequence | OF | up | up | yes |
| CB493454; CB510500; CA04317; CB492836; CB511307; CB497818; CB509992; CA045988 | Q01584 | Lipocalin precursor | OF | down | down | yes |
| CB496664 | Q9P2K5 | Myelin expression factor 2 | OF | down | down | yes |
| CB498057; CB516377; CA052634; CK991309; CB501115; CK990710; CB491722; CB497381; CB493362; CB497649 | P15880 | 40S ribosomal protein S2 | PS | up | up | yes |
| CA061291 | P46777 | 60S ribosomal protein L5 | PS | down | down | yes |
| CB497957 | P50894 | 40S ribosomal protein S7 | PS | up | up | yes |
| CA036950 | Q9UJW8 | Zinc finger protein 180 | PS | down | down | yes |
| CA048808 | AY785950 | Salmo salar zonadhesin-like gene, complete cds and 3' UTR | RPD | up | up | yes |
| CB503486; CB493928; CB494142; CK991176; CB498072 | Q9PVK2 | Alpha-enolase | UNKNOWN | up | up | yes |
| CA061664 |  | UNKNOWN | UNKNOWN | down | down | yes |
| CA048081 |  | UNKNOWN | UNKNOWN | up | up | yes |
| CB502026 |  | UNKNOWN | UNKNOWN | up | up | yes |
| CK991231 |  | UNKNOWN | UNKNOWN | down | down | yes |
| CK990510 |  | UNKNOWN | UNKNOWN | up | up | yes |
| CB500000 |  | UNKNOWN | UNKNOWN | down | down | yes |
| CB497128 |  | UNKNOWN | UNKNOWN | up | up | yes |
| CA037885 | O13085 | Cytochrome c oxidase polypeptide VIa, mitochondrial precursor | EM | up | down | no |
| CA056752 | P00940 | Triosephosphate isomerase | EM | up | down | no |
| CB498577; CB510792; CB509391; CB493676; CA038871; CB510589 | P28022 | Gamma crystallin M3 | EM | down | up | no |
| CB498361 | Q05025 | Glyceraldehyde-3-phosphate dehydrogenase | EM | up | down | no |
| CB496804 | Q6PBH5 | NADH dehydrogenase [ubiquinone] 1 alpha subcomplex subunit 4 | EM | up | down | no |
| CB496535; CA051403 | AB162342 | Oncorhynchus mykiss genes, MHC class I a region, complete and partial cds | IR | up | down | no |
| CB501671 | P62161 | Calmodulin | MCR | up | down | no |
| CB496805 | P68246 | Troponin I, fast skeletal muscle | MCR | up | down | no |
| CK991290; CB494318; CB493295 | Q90339 | Myosin heavy chain, fast skeletal muscle | MCR | down | up | no |
| CB493603; CB509706 | Q9I8V0 | Parvalbumin-2 | MCR | down | up | no |
| CB493603; CB509706 | Q9I8V0 | Parvalbumin-2 | MCR | down | up | no |
| CA038670; CB510556; CB510144; CB509947 | Q64438 | Angiogenin-2 precursor | PM | up | down | no |
| CB514790 | A0SXL6 | Elongation factor 2 | PS | down | up | no |
