# Supplementary material for: Divergence in gene regulation at young life history stages of whitefish (Coregonus sp.) and the emergence of genomic isolation
Source: BMC Evol Biol. 2009 Mar 16;9:59. doi: 10.1186/1471-2148-9-59 (PMC2662803; doi:10.1186/1471-2148-9-59)
Supplement: Additional file 3 — Regulatory changes between juvenile dwarf and normal whitefish at candidate adaptive traits. Comparison of significant regulatory changes between juvenile dwarf and normal whitefish with patterns observed at candidate adaptive traits. Derome et al. [20] and St-Cyr et al. [22] analysed gene expression in white muscle and liver tissue of adult fish from species pairs in natural lakes (dwarfs and normals from Cliff Lake and Indian Pond) and identified candidate adaptive traits based on patterns of parallel divergence. 96 EST clones that match candidate adaptive traits displayed significant patterns of divergence in whole juvenile dwarf and normal whitefish in this study (Lake Témiscouata "dwarf" and Lake Aylmer "normal" respectively). Column contents correspond with Additional file 2 [see Additional file 2], with an additional column describing regulatory changes found by St-Cyr et al. [22] for adult fish liver tissue and the following additional biological functions as described in St-Cyr et al. [22]: DT = detoxification; LM = lipid metabolism; BT = blood and transport; GLF = germ-line formation; PD = Protein degradation. A minus (-) indicates that gene expression divergence was not tested or detected in the corresponding adult tissue. Ten out of 26 genes clones show regulatory changes in the controlled environment that are congruent with the patterns observed in candidate adaptive traits in independent dwarf normal pairs in natural lakes. Eight of these ten genes are related to energy metabolism, which shows that regulatory changes between dwarf and normal whitefish related to this function are more constant across different environments, tissues and life history stages than those related to other functions. [file 1471-2148-9-59-S3.doc]

| **EST clone ID** | **Accession Number** | **Gene** | **Biological Function** | **Juvenile fish (whole)** | **Adult Muscle**  **[20]** | **Adult Liver**  **[22]** | **Congruent**  **changes** |
| --- | --- | --- | --- | --- | --- | --- | --- |
| CB496806; CA042792 | O13085 | Cytochrome c oxidase polypeptide VIa, mitochondrial precursor | EM | down | down | down | yes |
| CA039027; CA768062; CB491826; CB493574; CB491157; CB497681; CK991014; CB498361 | P00355 | Glyceraldehyde-3-phosphate dehydrogenase | EM | up | - | up | yes |
| CB514705; CB496739; CA062911; CB493709; CB517144 | P05065 | Fructose-bisphosphate aldolase A | EM | up | - | up | yes |
| CA057166; CB492725; CA041894; CB494346; CB509700 | P15429 | Beta-enolase | EM | up | up | - | yes |
| CB491722; CB497381; CB493362; CB497649; CB491722; CB497381; CB493362; CB497649; CB497649 | P19804 | Nucleoside diphosphate kinase B | EM | down | down | down | yes |
| CK990485 | P35031 | Trypsin-1 precursor | EM | up | - | up | yes |
| CB510934; CB492030; CB511030; CA042095; CB492512; CB510537; CB497378; CB493401; CB510934; CB492030; CB511030; CA042095; CB492512; CB510537; CB497378; CB493401 | Q05982 | Nucleoside diphosphate kinase A | EM | down | down | down | yes |
| BU965756; CB514460; CA050886; CB508064 | Q4KYY3 | Glyceraldehyde-3-phosphate dehydrogenase | EM | up | - | up | yes |
| CB496407 | P56533 | Betaine aldehyde dehydrogenase | DT | up | - | up | yes |
| CB504199; CK990712 | P80856 | Gastrotropin | LM | up | - | up | yes |
| CB509509; CA036673; CB501248 | P24774 | Plasma retinol-binding protein I | BT | down | - | up | no |
| CA054829 | Q56TU0 | Type-4 ice-structuring protein precursor | BT | down | - | up | no |
| CA037885 | O13085 | Cytochrome c oxidase polypeptide VIa, mitochondrial precursor | EM | up | down | down | no |
| CA056752 | P00940 | Triosephosphate isomerase | EM | up | down | - | no |
| CB508872; CB505763; CK990951 | P23591 | GDP-L-fucose synthetase | EM | down | - | up | no |
| CB498577; CB510792; CB509391; CB493676; CA038871; CB510589 | P28022 | Gamma crystallin M3 | EM | down | up | - | no |
| CB498361 | Q05025 | Glyceraldehyde-3-phosphate dehydrogenase | EM | up | down | up | no |
| CA061998 | P24723 | Protein kinase C eta type | GLF | down | - | up | no |
| CB497373; CB496526; CA039335 | P68246 | Troponin I, fast skeletal muscle | MCR | down | down | up | no |
| CB496805 | P68246 | Troponin I, fast skeletal muscle | MCR | up | down | up | no |
| CB493603; CB509706 | Q9I8V0 | Parvalbumin-2 | MCR | down | up | - | no |
| CB493454; CB510500; CA043176; CB492836; CB511307; CB497818; CB509992; CA045988; CB493454; CB510500; CA043176; CB492836; CB511307; CB497818; CB509992; CA045988 | Q01584 | Lipocalin precursor | OF | down | down | up | no |
| CK990562 | Q9GKL8 | Cathepsin L precursor | PD | down | - | up | no |
| CK991302 | P27797 | Calreticulin precursor | PS | up | - | down | no |
| CA063352 | P80311 | Peptidyl-prolyl cis-trans isomerase B precursor | PS | up | - | down | no |
| CK990518 |  | UNKNOWN | UNKNOWN | down | - | up | no |
